# Unveiling chemical industry secrets: Insights gleaned from scientific literatures that examine internal chemical corporate documents—A scoping review

**DOI:** 10.1371/journal.pone.0310116

**Published:** 2025-01-02

**Authors:** Miaoran Dong, Marc-André Gagnon

**Affiliations:** 1 School of Journalism and Communication, Carleton University, Ottawa, Canada; 2 School of Public Policy and Administration, Carleton University, Ottawa, Canada; Federal University of the ABC: Universidade Federal do ABC, BRAZIL

## Abstract

**Objective:**

Examine peer-reviewed scientific articles that used internal industry documents in the chemical sector to reveal corporate influence. Summarize sources of internal documents used in prior scientific papers to identify ongoing corporate strategies within the chemical field. Compare the corporate strategies identified in the chemical sector with the ones identified already identified in the pharmaceutical sector. Propose a theoretical framework for categorizing and examining the different form of corporate capture at play.

**Design:**

Performed a scoping review to pinpoint scientific papers employing internal industry/corporate documents within the chemical sector.

**Methods:**

We conducted a systematic search using broad and case study-derived keywords, detailed in the S1 Appendix. This resulted in 351 sources from 28 databases, encompassing peer-reviewed articles analyzing internal documents of chemical corporations. We complemented our efforts with a snowball sampling method to uncover additional case studies and journal articles not initially captured by our search. Results were categorized and analyzed using Marc-Andre Gagnon and Sergio Sismondo’s ghost management framework.

**Results:**

The final results included and analyzed 18 scientific papers. Legal proceedings served as the primary source of internal document data for all examined articles. We uncovered and categorized dynamic strategies employed by chemical corporations to protect and advance their interests, including scientific capture (n = 16), regulatory capture (n = 15), professional capture (n = 7), civil society capture (n = 6), media capture (n = 4), legal capture (n = 4), technological capture (n = 3), and market capture (n = 2).

**Comparative analysis:**

The limited scientific literature meeting our criteria confirms early findings by Wieland et al, highlighting a research gap in the chemical industry. Our analysis, building on the ghost-management framework, shows a different emphasis in the way internal documents were used in scientific literature to understand corporate strategies at play in the chemical sector as compared to the pharmaceutical sector. In contrast to Gagnon and Dong’s pharmaceutical corporate capture review, which identified 37 papers before 2022, our chemical industry findings reveal a lower count, with only 18 papers identified. Notably, the vast majority of the papers in both sectors shows an emphasis on analyzing strategies used for scientific capture. However, the area of regulatory capture reveals a significant distinction: only 6 of the 37 articles related to the pharmaceutical industry analyzed this dimension, as compared to 15 of the 18 articles related to the chemical industry. This body of work suggests that existing research on the chemical industry is particularly concerned with analyzing how the sector navigates and circumvents regulatory oversight. Both industries employ strategies involving conflicts of interest and the legitimization of their actions to shield themselves from public policy scrutiny and protect their interests. However, their goals seem to be significantly different. The scientific literature analyzing the pharmaceutical industry’s internal document tends to identify strategies maximizing profits through the biased promotion of health products, whereas the scientific literature analyzing the chemical industry’s internal documents is more inclined in identifying strategies institutionalizing ignorance about existing risks, evading accountability, and preventing regulatory actions.

**Strengths:**

Our scoping review shows how internal documents can reveal how the chemical industry strategically institutionalizes ignorance to manage business risks. It exposes intentional efforts by chemical corporations to promote ignorance and foster conflicts of interest, thereby legitimizing their business models and safeguarding corporate interests. We shared our research findings on the Dataverse/ Borealis platform (https://doi.org/10.5683/SP3/EOIOAU), making them accessible for future studies to apply the same analytical framework seamlessly.

**Limitations:**

We excluded papers that did not meet our research criteria, prioritizing those that analyzed internal corporate documents for uncovering covert ghost management captures. Beyond scientific literature, various grey literature sources have conducted quality investigations on ghost management strategies in the chemical industry, and many leaked internal documents in the chemical industry, often available through toxicdocs.org, were not analyzed in the scientific literature. Also, market concentration and other corporate captures can be investigated using publicly available resources. Despite searching scientific papers in various languages, no relevant publications were found outside of English. This presents an opportunity for future research to conduct a separate scoping review.

## Introduction

While chemicals can significantly improve lives and productivity, strategies used by chemical corporations to influence regulations and practices in order to increase profitability can negatively impact both human health and the environment. To better understand how corporate strategies can increase profitability to the detriment of public health, it is essential to investigate how scientific literature addresses corporate influence on shaping scientific knowledge, public policy discourse, and public discussions. No such systematic review exists to analyze ongoing problematic corporate strategies in the chemical sector. Internal corporate documents offer crucial insights into the strategies employed by corporations to influence scientific knowledge, regulation and practices. They often serve as unique and irreplaceable sources for evidence of corporate activities in pursuit of strategic goals [1–4]. These documents can shed light on the deliberate use of strategies detrimental to the public health for financial gain. Given that corporate interests have traditionally relied heavily on secrecy and that much of the information regarding potential risks of products is often treated as confidential business information, accessing and disclosing company documents, typically obtained through litigation, can be highly challenging [5].

We utilize the conceptual framework of ghost management as articulated by Marc-André Gagnon and Sergio Sismondo [6–8], as well as Ulrich Beck’s conceptualization of the risk society [9] to guide our analysis of corporate influences in the chemical industry. The notion of ghost management was developed by Sismondo to characterize the systematic utilization of tactics by pharmaceutical companies to mold medical knowledge and practices [7, 8, 10]. Gagnon extends this conceptualization to encompass corporate strategies that exert influence over other strategic aspects of business success such as market power, regulatory capture and technological path dependency [6, 11]. In alignment with Miller and Harkins’ delineation of four categories associated with corporate capture [12], originally devised for the alcohol lobby, Gagnon delineates seven distinct ghost management categories: 1) scientific capture (pertaining to the influence on knowledge production); 2) professional capture (concerned with the influence on healthcare practices); 3) technological capture (focused on steering technological pathways); 4) regulatory capture (centered on shaping laws to serve commercial interests); 5) market capture (aimed at establishing market dominance or constraining competition); 6) media capture (addressing the influence over media institutions); and 7) civil society capture (pertaining to the influence over charities, non-governmental organizations, trade unions, and civil society groups). Gagnon and Dong’s classification of ghost management capture encompasses seven categories, yet it might not cover all possible scenarios. Any corporate strategies not falling within these seven categories can be placed under an “other” category for further analysis. Diverging from Carpenter and Moss [13], our approach adopts a more expansive conceptualization of “capture,” denoting the objective of ghost management strategies without necessarily implying their absolute success.

Profitability of companies is traditionally seen as a sign of their good performance on markets, giving way to the suggestion that profits are compensation for a positive contribution to innovation, wealth or well-being. However, the concept of ghost management challenges this view, revealing how corporations deliberately and systematically intervene at different levels of societal structures to increase their profitability, often to the detriment of public health and welfare [6, 11]. Building on the works of Thorstein Veblen which consider capital a predatory force exerted on the social system [14, 15], the concept of ghost management allows a more refined understanding about the ways these forces are being deployed.

In particular, Ulrich Beck’s conceptualization of the risk society [9] can be helpful to understand these dynamics. For Beck, with the technological advances of the 20^th^ century, the economic system does not only produce “goods” (wealth and well-being), but it also produces “evils” (or risks). New products and innovation do not only contribute to well-being, but they also often come with potential risks and negative externalities. While “goods”, in their traditional sense, are self-evident (a physical product, or a service we benefit from), “evils” or “risks” are not. In order to exist, a risk must be determined through scientific research. Risks are not self-evident, they are social constructions; they must be defined, characterized and managed through the lenses of socio-political structures [9]: “While such things as income and education are consumable goods that can be experienced by the individual, the existence of and distribution of risks and hazards are mediated on principle through argument” (p. 27). Because many stakeholders can have interests in keeping some risks hidden, or in inflating the importance of other risks, the social existence (or not) and regulation (or not) of risks has less to do with scientific evidence and more to do with socio-political struggles [9]: “The social effect of risk definitions is not dependent on their scientific validity” (p. 32). The question of who decides what is or is not a risk becomes central, and the way risks are defined and managed reflects existing power relations. For Beck [16], risks “are products of struggles and conflicts over definitions within the context of specific relations of definitional power” (p. 30). Since, for specific products, the existence of risks is mediated through socio-political debates and arguments, the production of “good arguments” can become central for the business success and earning-capacity of corporations. As Beck predicted in 1988 (26): “Argumentation craftsmen have sunny days ahead” (p.32).

The concept of ghost management emphasizes the fact that businesses are not only invested in producing value or wealth, but also in producing arguments and habits of thought that will shape the social determinants of value [11]. The earning-capacity of the corporation depends less on the production of a product, than on the production of the belief that the product is necessary or safe, or on the production of favourable socio-economic institutions, such as regulations or dominant narratives, for their product. Ghost management can be used to conceal established risks, hinder the identification of new risks, and obstruct regulatory actions.

Taking inspiration from the pharmaceutical scoping review conducted by Gagnon and Dong in 2022 [17], our study performs a scoping literature review of previous scientific investigations that utilized internal chemical industry documents. This approach aims to shed light on hidden practices within corporations, fostering a better-informed public and supporting the implementation of effective regulations.

This scoping review investigates how chemical companies further their corporate interests by examining what the scientific literature has uncovered through the use of internal documents. In contrast to Wieland et al. [2], who primarily map scientific articles across various industries, our goal is more expansive. We seek to systematically categorize insights from these papers, identifying tactics and strategies. Additionally, we compare the previously completed pharmaceutical scoping review [17] with this new work on the chemical industry, highlighting parallels and distinctions in the corporate strategies of each sector that have been identified in the scientific literature.

This review marks the initial phase of a broader research endeavor, outlining corporate strategies in the chemical sector and establishing a framework for future studies in other industries. Our categorization and theorization framework for ghost management in the chemical industry will become a valuable tool for subsequent case studies.

## Methods

Scoping reviews offer a robust tool to comprehensively map existing literature, effectively summarize the scope of current studies, elucidate known findings, and identify areas that warrant further investigation [18, 19]. Our primary research for this scoping review centers on of internal corporate documents within scientific literature to investigate business strategies employed by chemical companies. To comprehensively explore this subject, we have delineated specific areas of inquiry:

1. **Articulation of Research Questions**: Scrutinizing the types of research questions posed in articles which make use of internal corporate documents.
2. **Methodological Approaches**: Examining the methodologies employed in studies using these internal documents.
3. **Scope of Investigation**: Identifying the specific products or substances that were the subjects of inquiry in these research endeavors.
4. **Influence Strategies**: Delving into the strategies adopted by these companies to influence scientific knowledge, professional practices, public policies, and public opinion.

Our goal is to build a comprehensive understanding of the way internal corporate documents have been used in academic research to illuminate diverse dimensions of chemical companies’ operations and ghost management strategies. Through revealing these dynamics, we seek to provide valuable insights into the intricate interplay between the chemical industry and the broader societal framework. Moreover, this research aims to pinpoint potential domains warranting regulatory and policy interventions to safeguard public interests.

### Criteria

In seeking scientific articles or book chapters that use internal corporate documents to reveal aspects of chemical companies’ operations and covert ghost management strategies, there were three main criteria for inclusion in our study: peer-reviewed status, explicit citation of internal company documents as the primary data source, and exploration of corporate captures within chemical companies.

We adhere to the internal document definition articulated by Gagnon and Dong [17], characterizing them as corporate/industry documents normally not accessible to the public, typically obtainable through court orders, leaks, or whistleblowers. Documents initially intended for public access, like those on corporate websites, were excluded. This broad definition was intentionally chosen to encompass various analyses related to chemical ghost management utilizing internal corporate/industry documents. Additionally, selected articles needed to examine one of the eight established ghost management strategies outlined in our theoretical framework or any new strategies employed by chemical companies.

### Extracting scientific articles

**Fig 1:**
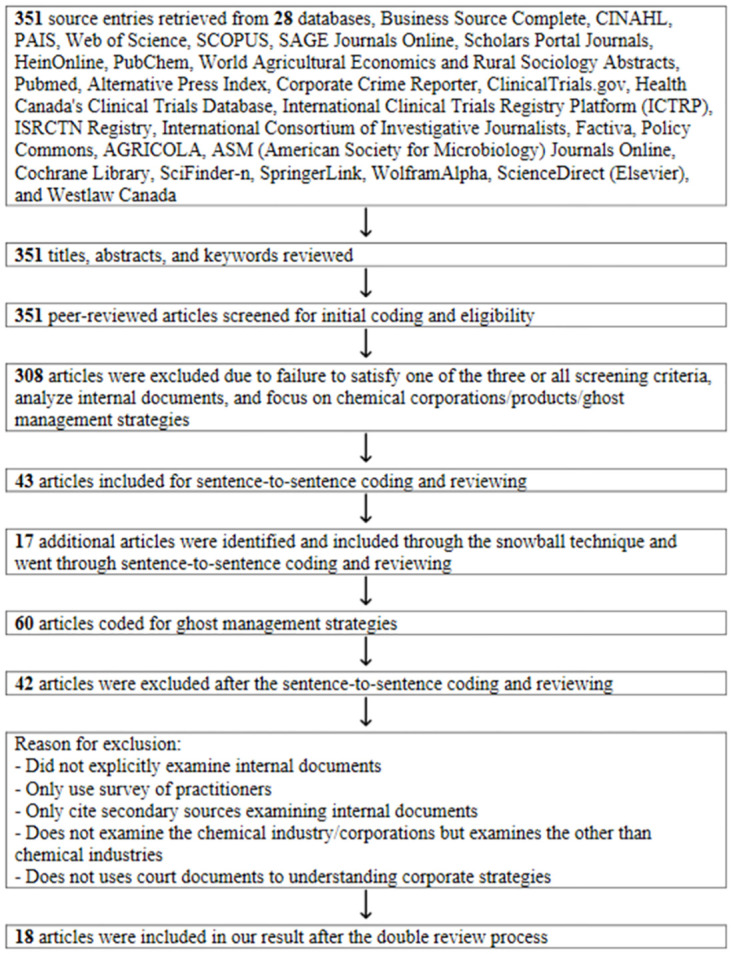
Flow chart of scoping review process.

In our search for scientific literature incorporating internal chemical corporate documents, we initially employed broad keywords across 28 databases (see S1 Appendix and Fig 1). We also used keywords from case studies of companies like Monsanto, Dow Agrosciences, Adama, Ciba-Geigy, Sandoz, Astra, ICI, Bayer, Schering, Hoechst, Rhone Poulenc, Rohms & Haas, Eli-Lilly, Dow Chemical, DuPont, BASF, ACC/American Home Products, American Home Products, Novartis, Astra-Zeneca, AgrEvo, Dow Chemical, Syngenta, Aventis CropScience, Bayer CropScience, Syngenta, ChemChina, Corteva, and BASF, where internal corporate documents were publicly available through litigations.

We extracted 351 academic papers with our search keywords and reviewed their titles, abstracts, and keywords to eliminate duplicates, excluding 308 articles that did not meet our screening criteria. We then performed detailed coding and screening on the remaining 43 articles. During this process, we identified 17 additional scientific articles using the snowball sampling method [20] to complement our search efforts. Subsequently, we coded 60 scientific articles to identify ghost management strategies employed by chemical companies.

### Coding

To conduct a comparative analysis with Gagnon and Dong’s pharmaceutical scoping review in 2022 [17], we employed the identical excel pivot table template available on the Dataverse (https://borealisdata.ca/dataverse/Ghost management). Our workbook has been made accessible on Dataverse to facilitate public access and enable future researchers to seamlessly apply the same analytical framework to their respective case studies (https://doi.org/10.5683/SP3/EOIOAU). Specifically, one team member manually entered coding information for 18 articles published between 2001 and 2024 [1, 21–37].

The workbook comprises 12 sheets, including a summary page with the corporate capture outline, explanations for eight ghost management captures based on our theoretical framework, a summary of case studies, sources for data collection, and an overview of research methodology. In the summary sheet, we denote the presence of each capture with Y or N. Additional concerns identified during separate coding by another researcher are documented in the MAG comments.

One team member initiated the scoping review search on December 19, 2022. To validate the results and capture new publications, another researcher conducted a matching scoping review search on May 13, 2024. All scientific articles extracted were published prior to May 13, 2024. Subsequently, one researcher coded all articles between February 1, 2023, and May 20, 2024, while another researcher conducted a separate coding on May 30, 2024. The two researchers engaged in discussions to resolve divergent coding decisions.

### Patient and public involvement

This study excluded patient inclusion or participation.

### Ethics statement

Ethical approval was unnecessary as the study did not entail the involvement of human participants or include research on animals.

## Results

Out of the 60 coded papers on chemical industry ghost management captures, 42 were excluded for various reasons (refer to Fig 1 for the search, code, and exclusion process, and S2 Appendix for the excluded articles). The exclusion criteria involved articles that didn’t explicitly examine internal documents, solely relied on practitioner surveys, cited secondary sources examining internal corporate documents, did not scrutinize the chemical industry/products directly, or failed to discover/examine covert corporate captures.

To ensure inclusion in the final results, articles underwent an independent review by two researchers, with inclusion contingent upon unanimous agreement that all search criteria were met. In cases of discrepancies during independent reviews, researchers collaboratively addressed them over a Zoom discussion. Ultimately, 18 scientific articles [1, 21–37] were included in the final analysis, synthesizing all ghost management chemical corporate captures from these articles in Table 1.

**Table 1:** Overview of strategies of captures in 18 articles.

Article	Regulatory	Scientific	Media	Market	Professional	Civil Society	Technological	Other
Nakajima, N. (2001).	Y	Y	Y	N	Y	Y	N	N
Weaver, C. K., & Motion, J. (2002)	Y	Y	Y	N	Y	Y	N	N
Epstein, S. S. (2005).	Y	Y	N	N	N	Y	N	N
Egilman, D. S., & Billings, M. A. (2005).	N	Y	N	N	N	N	N	Y
Egilman, D. S., Bird, T., & Lee, C. (2013).	Y	Y	N	Y	Y	Y	N	Y
Sarkar, S. (2014).	Y	N	N	N	N	N	N	N
Egilman, D., Bird, T., & Lee, C. (2014).	Y	Y	N	N	N	N	N	Y
Krimsky, S., & Gillam, C. (2018).	Y	Y	N	N	Y	N	N	N
Markowitz, G., & Rosner, D. (2018).	Y	Y	N	N	Y	Y	N	Y
McHenry, L. B. (2018).	N	Y	Y	N	Y	N	N	N
Richter, L., Cordner, A., & Brown, P. (2018).	Y	Y	N	N	N	N	N	N
Vainio, H. (2020).	Y	Y	N	N	N	N	N	N
Glenna, L., & Bruce, A. (2021).	Y	Y	N	Y	N	N	Y	N
Richter, L., Cordner, A., & Brown, P. (2021).	Y	Y	N	N	N	Y	Y	N
Bird, T., Steffen, J. E., Tran, T. H., & Egilman, D. S. (2021).	Y	Y	Y	N	Y	N	Y	N
Bacon, M.-H., Vandelac, L., Gagnon, M.-A., & Parent, L. (2023).	Y	Y	N	N	N	N	N	N
Rosner, D., & Markowitz, G. (2023).	N	Y	N	N	N	N	N	N
Messmer, M. F., Siegel, L., & Locwin, B. (2024).	Y	Y	N	N	N	N	N	N

### Case studies

Our curated scoping review includes articles focused on Monsanto, with seven discussing glyphosate-based herbicides, one on polychlorinated biphenyls (PCBs), and another on neonicotinoids [26–28, 33, 35, 36, 38]. Additionally, there are four articles on asbestos [21–24], three on per- and polyfluoroalkyl substances (PFAS) or Teflon [30, 31, 36], one on greenwashing [22], one analyzing McKinsey’s role in agribusiness transformation [32], one on GMO salmon [34], one on argi-chemical [29], and another shedding light on collaborative efforts between the United States and Dow to hinder European regulatory initiatives [25].

### Methods used

In contrast to the pharmaceutical industry scoping review, the examination of the chemical industry reveals a broader range of research methods. Notably, historical analysis, interviews, and archival research stand out as noteworthy methodologies, providing valuable supplements to theoretical frameworks. Richter et al’s literature review [30, 31] holds particular significance. All 18 papers employed qualitative analysis [1, 21–37]. Five articles exclusively utilized qualitative analysis, while the remaining 13 employed multiple methods, including content analysis [1, 25, 27–29, 32–37], historical analysis [25, 29], interview [30, 31], archival research [30, 31], and quantitative methods [32].

### Sources for internal industry documents

Among 18 scientific articles [1, 21–37], 15 used internal chemical corporate documents initially obtained via litigation [1, 21–28, 31–33, 35–37]. In contrast to pharmaceutical scoping reviews, our analysis indicates a trend of studies using leaked memos as their primary investigative source. Seven papers [23, 25, 29, 30, 32, 34, 36] employed leaked memos to examine chemical corporate captures, including personal communications, meeting minutes and speech transcripts.

15 papers used multiple sources in their investigations [1, 21–28, 31–33, 35–37]. Eight incorporated archival documents [1, 21, 27, 28, 28, 31–33], three examined corporate public documents like financial records and marketing materials [29, 34], while two relied on other academic literature as secondary sources [23, 32, 37]. One paper referenced policy documents [25], and another included interviews [31]. Krimsky and Gillam augmented their research with contributions from investigative journalists and legal professionals [1], suggesting alternative avenues for valuable insights beyond legal proceedings [38]. All resources and archival sites are documented in S3 Appendix for easy access in future research. We have compiled these channels for accessing internal documents from chemical companies to facilitate and encourage further research endeavors. Notably, ToxicDocs has aggregated a substantial collection of internal documents from various chemical companies. Three of our analyzed published articles have drawn directly from the internal documents available in toxicdots.org [27, 28, 38].

### Ghost management strategies in chemical industry

After analyzing 18 articles [1, 21–37], we identified eight distinct ghost management strategies employed by chemical companies to shape public perception. These strategies involve diverse methodologies aimed at influencing experts, manipulating regulatory frameworks, engaging with policymakers, and molding media and cultural narratives in alignment with corporate interests.

Table 1 summarizes the identified ghost management strategies from the analysis of these articles [1, 21–37]. The subsequent sections provide a comprehensive exploration of each strategy, offering specific examples and insights to enhance our understanding of how chemical companies wield influence. This detailed examination aims to underscore potential implications for public perception and decision-making.

### Scientific capture

16 scientific articles had a predominant focus on scientific capture, specifically examining how corporate funding in the chemical sector manipulates knowledge production [21–31, 33–37].

The most investigated aspect within scientific capture was the conflict of interest in chemical research, involving investigators, journals, and author interference, addressed in 14 articles [21–24, 26–28, 30, 31, 33–37]. Among these, seven articles delve into conflicts of interest with journalists and publishers [21–24, 26, 28, 34], four articles explore conflicts of interest with authors [23, 24, 28, 34–36], and five articles investigate conflicts of interest with investigators [21, 23, 24, 30, 34–36].

In addition, 11 articles explore non-disclosure and selective reporting, where unfavourable results were intentionally concealed [21–24, 26, 27, 30, 34–37], while eight highlight the prevalence of scientific articles written by ghostwriters paid by chemical corporations [23, 26, 28, 29, 34–37]. 11 articles investigate how the strategic downplaying of negative results reflects a deliberate effort to mitigate the perception of risks associated with products [21, 23–27, 30, 34–37]. Only one article delves into disease mongering in the chemical sector [34].

Among the 16 articles [21–31, 33–37], unique features of the chemical industry led to the creation of new subcategories for these captures. Nine articles examine how chemical corporations manufacture doubt [21–24, 28, 30, 31, 35, 36], while five study how these corporations discredit laypeople’s knowledge to undermine the reliability of research outcomes [21, 23, 24, 30, 31], and one paper focus on discrediting unfavorable researchers [35].

The overarching theme of scientific capture explores unseen and undone science, particularly the concealment of health effects within corporate-driven agendas. Tactics include efforts to augment corporate-interest favourable publications, suppress adverse findings, and silence unfavourable results.

### Regulatory capture

15 articles explored how chemical companies strategically influence or manipulate laws and regulations to prioritize corporate interests over public well-being [1, 21, 23, 25–27, 29–35, 37]. Employing various tactics, the articles show how companies established safety standards aligned with their interests, or create doubt and uncertainty over scientific evidence, effectively delaying legal proceedings, punishments, penalties or regulatory actions.

A prevalent pattern identified in 12 articles is the emergence of conflicts of interest with regulators, constituting a form of capture [1, 21, 23, 25, 26, 30–35, 37]. This capture dynamic is further examined in ten articles investigating lobbying and collaboration with trade associations as significant influencers of public policy [21–23, 25, 27, 29–31, 33, 37]. Examining the theme of self-regulation, six articles highlight how chemical companies resist external regulations [21, 23, 27, 31, 35, 37]. Furthermore, eight articles shed light on how chemical corporations collaborated with the US government to influence public policies in other countries [21–23, 30, 31, 35, 37]. Notably, Monsanto leveraged resources to depoliticize biotechnology, and in other cases, chemical companies collaborated with the American government to influence foreign regulations, employing misrepresentation and manipulation to hinder progress [25]. Lastly, a discernible pattern is the strategic targeting of regulators by chemical companies, tactically influencing decision-making processes. Collectively, these findings illuminate multifaceted and recurrent efforts by chemical companies to wield influence over laws, regulations, and policies, often at the expense of public health and safety.

### Professional capture

Seven articles reveal how chemical companies influence professionals to promote their products, cultivating intangible assets beneficial to corporate interests [1, 21, 23, 27–29, 34].

A recurrent strategy identified in the literature involves adapting messages and cultivating relationships—tailoring communications to resonate with professionals, and emphasizing product benefits while downplaying potential risks [23, 27, 34]. Simultaneously, articles explored how chemical corporations advised professionals in order to nurture relationships, fostering trust and loyalty [23, 29, 34]. Key opinion leaders played a crucial role, with four articles identifying influential professionals enlisted to endorse products or advocate for corporate interests, significantly amplifying credibility and acceptance [21, 28, 29, 34]. Moreover, three articles detail how chemical companies extend influence through training, education, and financing research initiatives, conferences, or educational programs to shape knowledge and practices aligning with corporate interests [1, 29, 34]. Additionally, one article highlights the practice of providing gifts and bribes [34], where companies offer incentives to professionals in return for support, compromising objectivity and undermining professional relationships. Another article explores the strategic dissemination of advertising materials directed at professionals as a tactic—leveraging diverse channels to directly promote products, fostering familiarity and preference [34].

Two articles investigate the discrediting of professionals [21, 29] and the issue of “hired guns” [21, 28]. Two articles also investigated smear campaigns employed to further discredit professionals [21, 28]. These observations highlight strategic maneuvers that have been employed by chemical companies to influence professionals, shaping perceptions and behaviors. Understanding these tactics fosters a nuanced recognition of potential conflicts of interest and biases within professional relationships and decision-making processes.

### Civil society capture

Six papers delve into the methods employed by chemical companies to sway various entities, including charities, NGOs, trade unions, social movements, and grassroots groups [23, 25, 27, 29, 31, 34]. Two papers specifically address conflicts of interest involving consumer groups [29, 34], be it through the establishment of front groups or through financial backing of existing groups.

Additionally, three papers shed light on how chemical companies dispute causation claims to evade compensating victims and their families. The articles detail how the companies successfully challenged established links between their products and adverse health effects, and ultimately sidestepped financial responsibility [23, 27, 31]. Furthermore, two papers reveal instances of chemical companies undermining opposing organizations [25, 29]. This included activities such as spying on environmental groups [25] and fabricating fake memos against environmental organizations [29]. These findings unveil intricate strategies that have been employed by chemical companies to influence a diverse range of groups. Through the use of front groups or financial support, they manipulated narratives, downplayed risks, and advanced their interests [23, 27].

### Media capture

Among the 15 papers analyzed [1, 21–34], four explored how chemical companies use media and communication strategies to shape public and “elite” or more informed opinions [21, 28, 29, 34]. These strategies involve targeted efforts and discrediting industry critics.

One significant aspect of media capture noted was direct-to-consumer advertising. Three articles examined how chemical companies engaged consumers directly through diverse marketing campaigns [3, 12, 17]. For example, one article explored the push to replace the phrase "talc used in cosmetics" with "cosmetic talc", which highlights the latter as a marketing construct [21]. Moreover, two papers examined how chemical companies collaborated with journalists and fostered conflicts of interest, thereby exerting influence on information production [28, 34], such as an individual affiliated with Forbes employed by Monsanto. An analysis of these dynamics provides a nuanced understanding of how information is produced and disseminated, with potential influences from corporate interests.

### Technological capture

Three articles delved into how chemical companies strategically use technological standards and the safeguarding confidential business information (CBI) to exert influence [21, 26, 31]. One article explores strategic patenting [26], while two others focus on the challenges posed by confidential business information [21, 31].

For instance, one article found that when chemical manufacturers label data as CBI, it becomes challenging for Environmental Protection Agency (EPA) scientists to access these data for research studies [31]. Manufacturers can claim submitted information as CBI, limiting its availability to designated EPA offices and approved staff, with strict regulations on sharing within the agency or other government entities [31]. Industries incur no expenses when designating information as confidential business information (CBI), and there is limited oversight or penalties for improper claims. However, as the article notes, the repercussions for mishandling or disclosing CBI data are severe, leading to criminal imprisonment and substantial fines [31].

Considering that transforming agriculture in agribusiness can lead to significant corporate control over the intellectual property in the technology involved in agribusiness, one article included in our review detailed how the formal endorsement of the Trade-related Aspects of Intellectual Property Rights (TRIPS) Agreement by the World Trade Organization (WTO) and national-level policies like the Plant Variety Protection Act in the United States confer exclusive proprietary rights to breeders [32].

### Market capture

Two papers [23, 26], investigate how chemical companies gain market power, restrict competition, and establish dominance. One paper focuses on market concentration in the chemical sector [26], revealing a significant link between primary seed transactions and the use of Monsanto’s Roundup herbicide. The other paper explores conflicts of investment [23], exemplified by MetLife’s investments in mining companies producing asbestos and silicosis products. Through detailed scrutiny of annual reports obtained via litigation, this article highlights financial ties between MetLife and the chemical industry. Understanding these investment patterns provides insights into how chemical companies can fortify their market positions.

### Other capture

Four articles explore other captures [22–24, 27], with one focusing on the deliberate and unlawful actions of chemical corporations [27]. Three articles explore previously unidentified legal captures [22–24], with one of these articles detailing how industry professionals redefined evidentiary standards for proving causation in potential carcinogen cases [24]. The article shows how asbestos corporations argued for stringent documentation in epidemiologic studies, emphasizing the need for clear evidence regarding the type of asbestos and the precise nature of exposure to establish causation in cancer development. Another article discussed corporate strategies including influencing worker compensation laws and establishing arbitrary protective standards for monitoring asbestos exposure, which facilitates the dismissal of sick workers without informing them of the results [23]. Additionally, collaborations with entities like MetLife, the Federal Bureau of Mines, and asbestos corporation maintain confidentiality of clinical findings.

All corporate captures identified in other categories involved strategic deployment of judicial resources, using existing laws and regulations to impose specific perspectives and actions over the judicial process itself. There is a pressing need for more research on legal capture as a self-contained category for ghost management, particularly concerning the burden of proof. This would refine and enhance our understanding of the Ghost Management framework.

## Comparative analysis with the pharmaceutical sector

In contrast to Gagnon and Dong’s scoping review on pharmaceutical corporate capture [17], which identified 37 peer-reviewed papers before 2022, our examination of the chemical industry reveals only 18 relevant papers. The scholarly examination of the pharmaceutical and chemical industries reveals a diverse and vibrant landscape of research on different aspects of industry capture. Specifically, 28 academic articles have explored scientific capture within the pharmaceutical sector, while 16 have focused on this issue within the chemical industry. Similarly, professional capture has been addressed in 16 articles related to pharmaceuticals and 7 related to chemicals. Market capture has attracted scholarly attention in 4 studies on the pharmaceutical industry and 2 on the chemical industry. The analysis of civil society capture has 4 studies on the pharmaceutical sector and 6 on the chemical sector. Media capture has been examined in 3 studies on pharmaceuticals and 4 on chemicals, while another set of studies shows 2 articles on media capture in the pharmaceutical industry and 3 in the chemical industry.

Notably, the vast majority of the papers in both sectors shows an emphasis on analyzing strategies used for scientific capture. However, the area of regulatory capture reveals a significant distinction: only 6 of the 37 articles related to the pharmaceutical industry analyzed this dimension, as compared to 15 of the 18 articles related to the chemical industry. This body of work suggests that existing research on the chemical industry is particularly concerned with analyzing how the sector navigates and circumvents regulatory oversight. Both industries employ strategies involving conflicts of interest and the legitimization of their actions to shield themselves from public policy scrutiny and protect their interests. However, their goals seem to be significantly different. The scientific literature analyzing the pharmaceutical industry’s internal document tends to identify strategies maximizing profits through the biased promotion of health products, whereas the scientific literature analyzing the chemical industry’s internal documents is more inclined in identifying strategies institutionalizing ignorance about existing risks, evading accountability, and preventing regulatory actions.

Adding complexity to this comparison, in contrast to the pharmaceutical scoping review, the papers from the chemical sector included in our review explore the way the concept of confidential business information (CBI) is constantly employed to further reduce access to scientific research and data. This approach poses a significant challenge for independent researchers and regulatory bodies, impeding access to data and hindering transparency in evaluating the safety and efficacy of chemical products.

## Discussion

Our research underscores how the use of the ghost management framework allows the identification of systematic corporate strategies, revealing pervasive conflicts of interest (COIs), legitimization tactics, and their consequential impact on the productions of knowledge and of ignorance within the chemical sector.

### A complex web of COIs

The identified strategies employed by chemical companies to manage their public image involves a complex web of conflicts of interest. Companies strategically nurtured conflicts of interest with various entities such as public institutions, regulatory bodies, professionals, media organizations, and scientific researchers, as revealed in our comprehensive review of peer-reviewed scientific literature [1, 26, 28, 33]. Internal corporate documents from the chemical sector reveals the significant impact these COIs can have on the definition of risks, on policy discussions, on public health and on commercial success.

A prime example of the pervasiveness of COIs is the analysis of the “Monsanto papers”, which shows how Monsanto (now part of Bayer) has promoted glyphosate-based herbicides (GBH). Internal corporate documents show that Monsanto fostered COIs with an impressive network of “independent” academics, scientific journal editors, journalists, regulators and consultants. Working with “independent experts”, Monsanto ghostwrote an important part of the existing literature about the safety of GBH, which has furthered arguments against constraining regulations [1, 26, 28]. Journal editors who also had COIs helped Monsanto interfere in the peer-review process of scientific papers [26, 28]. Monsanto also mobilized its network of “independent researchers” to successfully lobby journals for the retraction of scientific papers questioning the safety of GBH [1, 26, 28]. The company also ghostwrote in the lay media with the help of journalists attacking independent researchers questioning the safety of GBH [28]. Finally, internal corporate documents show Monsanto used their financial relations with three Environmental Protection Agency (EPA) officials to exert undue influence over the EPA and derail a safety review of GBH [1]. This state of affairs should not be surprising considering that Monsanto is well-known for its intimidation and attacks on the reputation of any scientist or journalist questioning the safety of their products [35]. The evidence of COIs from the Monsanto papers shows how the extent of corporate power in shaping narratives and practices, must not be underestimated.

### Ignorance/knowledge production

The manipulation of knowledge production and, in particular, the hiding and downplaying of data which may harm a company’s image or profitability seem to be central features of ongoing ghost management strategies related to scientific, professional and civil society captures in the chemical industry. Identified key tactics include non-disclosure of negative findings, systematic opacity of risks through CBI, downplaying of risks, selective reporting of data, ghostwriting of biased reports, bullying and discrediting “hostile” experts, and discrediting laypeople’s experience and knowledge of the risks and harms in using specific chemical products. In many ways, in the analyzed cases, it seems that the selective production of ignorance was as central to the business success of chemical corporations as the production of chemical products themselves.

When analyzing the regulatory structure for PFAS, Richter et al. [31] introduce the concept of the "institutionalized ignorance regime" in the chemical industry. While the concept is used to characterize the lack of regulation for PFAS, it also works well to describe some of the results in this scoping review. The "institutionalized ignorance regime" includes three levels of ignorance, each playing a significant role in shaping the industry’s practices and outcomes.

The first level is "selective ignorance", which involves deliberate efforts to limit (non-disclosure) and manipulate information (ghostwriting) in a way that creates doubt and helps companies evade regulatory scrutiny [29]. Selective ignorance could be compared to manufacturing doubt. By carefully controlling the flow of information, the industry attempts to shape public perception and avoid taking responsibility for potential risks associated with their products [29].

The second level is "forbidden knowledge", which refers to various practices that prevent certain information from becoming widely known [29]. As previously mentioned, the concept of confidential business information (CBI) serves as a means for companies to withhold important data from public scrutiny; the lack of resources within regulatory agencies can prevent a thorough analysis of health and safety data. Additionally, grandfather clauses in regulations, as seen in the Toxic Substance Control Act, provide exemptions to certain chemicals, allowing companies to bypass rigorous evaluation requirements.

The third level of ignorance, "nescience", refers to a complete lack of knowledge or awareness, which can lead to unforeseen risks and uncertainties. In the chemical industry, these risks are often treated as radical uncertainties, meaning they are not fully understood or adequately managed. By not doing the appropriate research on risks, these potential risks disappear as “unknown unknowns”, which allows the shifting of these risks onto civil society stakeholders, including workers, communities, researchers, and decision-makers, without sufficient risk management measures or precautionary actions [31].

Applying Beck’s [16] understanding that risks, as social constructions, are products of power struggle over definitions and are managed through socio-political structures it can be argued that the proposed notions of “selective ignorance” and “forbidden knowledge” [29] can describe dynamics which shape the ways risks are defined and managed. “Nescience” [29], however, refers to dynamics in which the research to find and define risks will not be done, creating radical uncertainty.

This scoping review, which investigated how chemical companies further their corporate interests by examining what the scientific literature has uncovered through the use of internal documents, re-enforces Richter et al.’s notion of the existence of an institutionalized multi-level ignorance regime. Addressing these manifestations of ignorance is essential for fostering a more enlightened, accountable, and responsible chemical industry that would better protect the well-being and safety of citizens.

## Limitations

We focused our research on analyzing internal corporate documents to unveil covert ghost management captures, excluding papers that did not meet this criterion. However, it is crucial to emphasize that the examination of corporate ghost management strategies should not be solely limited to internal corporate documents. Research involving public corporate documents, using tools like the Wayback Machine and other publicly available resources, can provide valuable insights as well [39, 40].

We exclusively focused on English-language scientific papers in our search, potentially excluding relevant articles lacking English translations due to our choice of keywords. Our investigation, centered on peer-reviewed scientific literature, English-centric databases, and examination of internal chemical corporate documents, lays a foundational but limited groundwork for comprehending corporate influence on scientific research, norms, and the chemical industry.

We did not directly analyze internal documents from chemical companies in this study. Instead, the primary aim of this article is to systematically review academic research that utilizes internal chemical company documents, in order to identify channels and methodologies for accessing these documents, thereby encouraging further research. Resources like ToxicDocs.org and the "BP Papers" recently released by The Downs Law Group at https://downslawgroup.com/bp-papers/ represent valuable repositories that require more comprehensive future case studies by scholars. Such analyses could further uncover the strategies employed by chemical companies to influence public policy and values.

By scrutinizing scientific literature which makes reference to internal corporate documents and categorizing these findings using ghost management categories, we gain deeper insights into the pervasive influence of chemical corporations. However, for a more comprehensive understanding, future research should extend beyond peer-reviewed literature to directly examine internal documents and incorporate diverse and multilingual sources such as journalistic investigations, criminal probes, or regulatory examinations. To enhance this analysis of the chemical sector, there is a need for further research to delineate ghost management categories, contributing to a more nuanced understanding of prevailing norms.

## Conclusion

Analyzing scientific literature which references internal company documents based on categories of ghost management allows a better understanding of how pervasive and “normal” corporate influence on knowledge and practices relating to chemicals has become. However, the limited scientific literature meeting our search criteria echoes a broader scarcity outlined by Wieland et al. [2]. This underscores the deficiency of this kind of research within the chemical industry and emphasizes the crucial necessity to scrutinize internal corporate documents.

Overcoming transparency challenges is crucial in enhancing public awareness and promoting scholarly investigations. To tackle this urgent concern, we must take decisive actions to dismantle institutionalized ignorance regimes that allow the chemical industry to avoid accountability. Prioritizing transparency, supporting independent research, and allowing informed public debates are crucial measures to mitigate this corporate power in order to better identify real risks and safeguard public interests. To be part of the solution, our research is publicly available on Dataverse (https://doi.org/10.5683/SP3/SQQJCA) to support future studies applying the same analytical framework to their investigations. Hopefully, it will contribute to a more sustainable, transparent, and responsible paradigm in chemical regulation and make it easier for other research to adopt the same investigational framework.

## Supporting information

S1 Appendix Keywords. (DOCX)

S2 Appendix A list of excluded articles. (DOCX)

S3 Appendix Data collection resources. (DOCX)
