# Supplementary material for: Unveiling chemical industry secrets: Insights gleaned from scientific literatures that examine internal chemical corporate documents—A scoping review
Source: PLoS One. 2025 Jan 2;20(1):e0310116. doi: 10.1371/journal.pone.0310116 (PMC11694964; doi:10.1371/journal.pone.0310116)
Supplement: S1 Appendix — (DOCX) [file pone.0310116.s001.docx]

# **Appendix 1 Keywords**

**Broad Set of Keywords**

("agriculture" OR “agrichemical” OR “agrochemical” OR “agro-chemical” OR “agro chemical” OR “agri chemical” OR “agri-chemical” OR “agriculture corporate” OR "agricultural industry" OR “insecticides” OR “insecticide” OR “herbicides” OR “herbicide” OR “fungicides” OR “fungicide” OR “pesticides” OR “pesticide” OR “nanoparticles” OR “nanoparticle” OR “glyphosate” OR “glyphosates” OR “co-formulants” OR “co-formulant” OR “adjuvant” OR “adjuvants”) AND ("internal document" OR "confidential document" OR "court document" OR "corporate document" OR "internal documents" OR "confidential documents" OR "court documents" OR "corporate documents" OR "leaked document" OR "leaked documents" OR “data disclosure” OR “confidential business” OR “corrupt” OR “whistleblower” OR “under report” OR “underreport” OR “watchdog” OR “Inquiry” OR “Litigation Discovery documents” OR “Litigation document” OR “Discovery documents” OR “Discovery document”)

**Keywords Derived from Case Studies**

(“Monsanto” OR “Dow Agrosciences” OR “Adama” OR “Ciba-Geigy” OR “Sandoz” OR “Astra” OR “ICI” OR “Bayer” OR “Schering” OR “Hoechst” OR “Rhone Poulenc” OR “Rohms & Haas” OR “Eli-Lilly” OR “Dow Chemical” OR “DuPont” OR “BASF” OR “ACC/American Home Products” OR “American Home Products” OR “Novartis” OR “Astra-Zeneca” OR “AgrEvo” OR “Dow Chemical” OR “Syngenta” OR “Aventis CropScience” OR “Bayer CropScience” OR “Syngenta” OR “ChemChina” OR “Corteva” OR “BASF”) AND ("internal document" OR "confidential document" OR "court document" OR "corporate document" OR "internal documents" OR "confidential documents" OR "court documents" OR "corporate documents" OR "leaked document" OR "leaked documents" OR “data disclosure” OR “confidential business” OR “corrupt” OR “whistleblower” OR “under report” OR “underreport” OR “watchdog” OR “Inquiry” OR “Litigation Discovery documents” OR “Litigation document” OR “Discovery documents” OR “Discovery document”)
