# Supplementary material for: Unveiling chemical industry secrets: Insights gleaned from scientific literatures that examine internal chemical corporate documents—A scoping review
Source: PLoS One. 2025 Jan 2;20(1):e0310116. doi: 10.1371/journal.pone.0310116 (PMC11694964; doi:10.1371/journal.pone.0310116)
Supplement: S2 Appendix — (DOCX) [file pone.0310116.s002.docx]

# **Appendix 2 A List of Excluded Articles**

1. Acquavella J, Burns C, Flaherty D, Holsapple M, Kimber I, Ladics G, et al. A Critique of the World Resources Institute’s Report “Pesticides and the Immune System: The Public Health Risks.” Environmental Health Perspectives. 1998;106(2):51–4.

2. Bhardwaj G, Camillus JC, Hounshell DA. Continual Corporate Entrepreneurial Search for Long-Term Growth. Management Science. 2006 Feb;52(2):248–61.

3. C C, Surgan M. Unidentified inert ingredients in pesticides: implications for human and environmental health. ENVIRON HEALTH PERSPECT. 2006 Dec;114(12):1803–6.

4. Castleman B. Asbestos Products, Hazards, and Regulation. Int J Health Serv. 2006 Apr 1;36(2):295–307.

5. Creager ANH. To Test or Not to Test: Tools, Rules, and Corporate Data in US Chemicals Regulation. Science, Technology, & Human Values. 2021 Sep 1;46(5):975–97.

6. Cseres KJ. “Acceptable” Cartels at the Crossroads of EU Competition Law and the Common Agricultural Policy: A Legal Inquiry into the Political, Economic, and Social Dimensions of (Strengthening Farmers’) Bargaining Power. Antitrust Bulletin. 2020 Sep;65(3):401–22.

7. Egilman D, Bird T, Wilson R. Use of Anti-Warnings to Falsely Reassure Downstream Users: An Asbestos Example. New Solut. 2018 Nov 1;28(3):515–38.

8. Egilman DS, Ardolino EL, Howe S, Bird T. Deconstructing a State-of-the-Art Review of the Asbestos Brake Industry. New Solut. 2012 Feb 1;21(4):545–71.

9. Fernández Ríos D, Rubinstein C, Vicién C. Capacities for the Risk Assessment of GMOs: Challenges to Build Sustainable Systems. Frontiers in Bioengineering and Biotechnology. 2018;6:40–40.

10. Halabi SF. Off-Label Marketing’s Audiences: The 21st Century Cures Act and the Relaxation of Standards for Evidence-Based Therapeutic and Cost-Comparative Claims. American journal of law & medicine. 2018;44(2–3):181–96.

11. Kleinman DL, Suryanarayanan S. Dying Bees and the Social Production of Ignorance. Science, Technology, & Human Values. 2013 Jul 1;38(4):492–517.

12. Kleinman DL, Suryanarayanan S. Ignorance and industry: Agrichemicals and honey bee deaths. In: Routledge International Handbook of Ignorance Studies. Routledge; 2015.

13. Knoeber CR. Explaining state bans on corporate farming. Economic Inquiry. 1997 Jan;35(1):151.

14. Lamphere JA, East EA. Monsanto’s Biotechnology Politics: Discourses of Legitimation. Environmental communication. 2017;11(1):75–89.

15. Lutter R, Barrow C, Borgert CJ, Conrad Jr. JW, Edwards D, Felsot A. Data Disclosure for Chemical Evaluations. ENVIRON HEALTH PERSPECT. 2013 Feb;121(2):145–8.

16. Machluf Y, Yarden A. Integrating bioinformatics into senior high school: design principles and implications. Briefings in Bioinformatics. 2013 Sep;14(5):648–60.

17. Marcoux C, Urpelainen J. Special Interests, Regulatory Quality, and the Pesticides Overload. Review of Policy Research. 2011 Nov;28(6):585–612.

18. Moyo D, Zungu M, Kgalamono S, Mwila CD. Review of Occupational Health and Safety Organization in Expanding Economies: The Case of Southern Africa. Ann Glob Health. 2015 Aug;81(4):495–502.

19. Murphy C. Competitive intelligence: What corporate documents can tell you. Business Information Review. 2006 Mar 1;23(1):35–42.

20. Neog Y, Gaur AK. Shadow economy, corruption, and tax performance: A study of BRICS. Journal of Public Affairs (14723891). 2021 May;21(2):1–7.

21. Panda A. Do You Know Who You Are Dealing With?Cultural Due Diligence: What, Why and How. Vikalpa. 2013 Apr 1;38(2):1–20.

22. Peekhaus W. Biowatch South Africa and the challenges in enforcing its constitutional right to access to information. Government Information Quarterly. 2011 Oct;28(4):542–52.

23. Ranald P. The Trans-Pacific Partnership Agreement: Reaching behind the border, challenging democracy. The Economic and Labour Relations Review. 2015 Jun 1;26(2):241–60.

24. Rathod SK. Patent linkage and data exclusivity: a look at some developments in India. Journal of Generic Medicines. 2011 Jul 1;8(3):140–9.

25. Roberts I, Bunn F. Egg on their Faces: The Story of Human Albumin Solution. Eval Health Prof. 2002 Mar 1;25(1):130–8.

26. Rüegger M, Schütz R. Medico-Legal Aspects of Asbestos-Induced Pleural Plaques: The Issue of Worker’s Compensation. Indoor + built environment. 1997;6(2):119–24.

27. Saenger C, Torero M, Qaim M. Impact of Third-party Contract Enforcement in Agricultural Markets—A Field Experiment in Vietnam. American Journal of Agricultural Economics. 2014 Jul;96(4):1220–38.

28. Schleifer D. We Spent a Million Bucks and Then We Had To Do Something: The Unexpected Implications of Industry Involvement in Trans Fat Research. Bulletin of Science, Technology & Society. 2011 Dec 1;31(6):460–71.

29. Schleifer D, Penders B. Food, Drugs, and TV: The Social Study of Corporate Science. Bulletin of Science, Technology & Society. 2011 Dec 1;31(6):431–4.

30. Skydan O, Yatsenko O. The Institutional Bases of Formation of Competitive Relations in Beekeeping: INSTITUCINIAI PAGRINDAI FORMUOJANT KONKURENCINIUS SANTYKIUS BITININKYSTĖJE. Management Theory & Studies for Rural Business & Infrastructure Development. 2010 Dec;23(4):118–25.

31. Smith RJN, Glegg GA, Parkinson R, Richards JP. Evaluating the implementation of the Nitrates Directive in Denmark and England using an actor-orientated approach. European Environment: The Journal of European Environmental Policy (Wiley). 2007 Mar;17(2):124–44.

32. Smith R, McElwee G. The “horse-meat” scandal: illegal activity in the food supply chain. Supply Chain Management. 2021 Sep;26(5):565–78.

33. Suryanarayanan S, Kleinman DL. Disappearing bees and reluctant regulators. Issues in Science and Technology. 2011 Jun 22;27(4):33–7.

34. Vosko LF. Tenuously Unionised: Temporary Migrant Workers and the Limits of Formal Mechanisms Designed to Promote Collective Bargaining in British Columbia. Industrial Law Journal. 2014 Dec;43(4):451–84.

35. Waldman P, Stecker T, Rosenblatt J. Guess Who’s Ghostwriting Monsanto’s Safety Reviews. Bloomberg Businessweek. 2017 Aug 14;(4534):14–5.

36. Washburn R. Conceptual Frameworks in Scientific Inquiry and the Centers for Disease Control and Prevention’s Approach to Pesticide Toxicity (1948–1968). American Journal of Public Health. 2019 Nov;109(11):1548–56.

37. Wegman DH. Learning from history. J Public Health Pol. 2018 Nov;39(4):459–62.

38. Xue C, Tang L, Walters D. Decoupled implementation? Incident reporting in Chinese shipping. Economic and Industrial Democracy. 2021 Feb 1;42(1):179–97.

39. Yarrington D. Cattle, Corruption, and Venezuelan State Formation During the Regime of Juan Vicente G&oacute;mez, 1908-35. Latin American Research Review. 2003 Apr;38(2):3–33.

40. Кирилов ЮЄ, Ігнатенко ММ, Грановська ВГ. Соціально-економічні чинники академічної мобільності у формуванні конкурентоспро-можності представників науково-освітньої спільноти: Socio-economic factors of academic mobility in the formation of competitiveness of representatives of a scientific and educational society. Социально-экономические факторы академической мобильности в формировании конкурентоспособности представителей научно-образовательного сообщества. 2021 Dec;(12):73–80.
