# Supplementary material for: Unveiling chemical industry secrets: Insights gleaned from scientific literatures that examine internal chemical corporate documents—A scoping review
Source: PLoS One. 2025 Jan 2;20(1):e0310116. doi: 10.1371/journal.pone.0310116 (PMC11694964; doi:10.1371/journal.pone.0310116)
Supplement: S3 Appendix — (DOCX) [file pone.0310116.s003.docx]

# **Appendix 3. Data Collection Resources**

- FDA documents released in response to Freedom of Information Act requests.” (Bird et al., 2021, p. 152)
- McKinsey documents (30)
- The Monsanto Papers (McHenry, 2018; Vainio, 2020)
- [David Egilman](https://repository.library.brown.edu/) Papers (Bird, 2021)
- [ToxicDocs](http://www.ToxicDocs.org) (25,26,33)
- [UCSF Industry Documents Library](https://www.industrydocumentslibrary.ucsf.edu/)
- [Baum](https://www.wisnerbaum.com/) Hedlund Aristei & Goldman national civil trail lawyers
- [COPE](https://publicationethics.org/files/retraction%20guidelines.pdf) (Committee on Publication Ethics) (33)
- United States (US) Environmental Protection Agency (EPA) through Freedom of Information Act (FOIA) (33)
- [U.S. Right to Know](https://usrtk.org/about/;%20https:/usrtk.org/pesticides/mdl-monsanto-glyphosate-cancer-case-key-documents-analysis/): (26,33);
- [Publicly accessible digital repository](https://usrtk.org/pesticides/mdlmonsanto-glyphosate-cancer-case-key-documents-analysis/): (33)
- [Glyphosate issue paper](https://www.epa.gov/sites/production/files/2016-09/documents/glyphosate%20_issue_paper_evaluation_of_carcincogenic_potential.pdf): evaluation of carcinogenic potential, September 12, 2016. (33)
- [Le Monde](https://www.lemonde.fr/) (33)
- [law firm of Baum, Hedlund, Aristei & Goldman](https://www.baumhedlundlaw.com/toxic-tortlaw/monsanto-roundup-lawsuit/monsanto-secret-documents/) (26)
- [Chemical Industry Documents](https://www.industrydocuments.ucsf.edu/results/#q=monsato&col=%5B%22bvhp%22%2C%22benzene%22%2C%22marketpr%22%2C%22nytepa%22%2C%22pfas%22%2C%22roundup%22%2C%22usrtk%22%2C%22sanjour%22%2C%22actos%22%2C%22blood%22%2C%22celexa%22%2C%22cochrane%22%2C%22cymbalta%22%2C%22neurontin%22%2C%22norvir%22%2C%22pogo%22%2C%22paxil%22%2C%22payments%22%2C%22pinnacle%22%2C%22prempro%22%2C%22risperdal%22%2C%22seroquel%22%2C%22vioxxlit%22%2C%22vioxxmar%22%2C%22zyprexa%22%5D&h=%7B%22hideDuplicates%22%3Afalse%2C%22hideFolders%22%3Atrue%7D&cache=true&count=16) (34)
